# Human Cytomegalovirus Infection Suppresses CD34^+^ Progenitor Cell Engraftment in Humanized Mice

**DOI:** 10.3390/microorganisms8040525

**Published:** 2020-04-06

**Authors:** Lindsey B. Crawford, Rebecca Tempel, Daniel N. Streblow, Andrew D. Yurochko, Felicia D. Goodrum, Jay A. Nelson, Patrizia Caposio

**Affiliations:** 1 Vaccine and Gene Therapy Institute, Oregon Health & Science University, Beaverton, OR 97006, USA; crawforl@ohsu.edu (L.B.C.); beckie.tempel@gmail.com (R.T.); streblow@ohsu.edu (D.N.S.); nelsonj@ohsu.edu (J.A.N.); 2 Department of Microbiology and Immunology, Louisiana State University Health Sciences Center, Shreveport, LA 71130, USA; ayuroc@lsuhsc.edu; 3 Department of Immunobiology, BIO5 Institute, University of Arizona, Tucson, AZ 85719, USA; fgoodrum@email.arizona.edu

**Keywords:** human cytomegalovirus, progenitor cell, hematopoietic stem cell transplant, myelosuppression, hematopoiesis, humanized mice

## Abstract

Human cytomegalovirus (HCMV) infection is a serious complication in hematopoietic stem cell transplant (HSCT) recipients due to virus-induced myelosuppression and impairment of stem cell engraftment. Despite the clear clinical link between myelosuppression and HCMV infection, little is known about the mechanism(s) by which the virus inhibits normal hematopoiesis because of the strict species specificity and the lack of surrogate animal models. In this study, we developed a novel humanized mouse model system that recapitulates the HCMV-mediated engraftment failure after hematopoietic cell transplantation. We observed significant alterations in the hematopoietic populations in peripheral lymphoid tissues following engraftment of a subset of HCMV^+^ CD34^+^ hematopoietic progenitor cells (HPCs) within the transplant, suggesting that a small proportion of HCMV-infected CD34^+^ HPCs can profoundly affect HPC differentiation in the bone marrow microenvironment. This model will be instrumental to gain insight into the fundamental mechanisms of HCMV myelosuppression after HSCT and provides a platform to assess novel treatment strategies.

## 1. Introduction

Human cytomegalovirus (HCMV) infection after Hematopoietic Stem Cell Transplantation (HSCT) is still a significant cause of morbidity and mortality in the transplant population. The spectrum of CMV infection can go from essentially asymptomatic (reactivation without any organ involvement) to CMV end-organ diseases including esophagitis, gastroenteritis, hepatitis, retinitis, pneumonia, and encephalitis. In addition, CMV infection can result in severe pancytopenia, myelosuppression, graft failure, and immunosuppression that may lead to the development of concurrent infectious complications [1]. Despite the well-described clinical relevance of hematopoietic lineage infection by HCMV, the fundamental mechanisms underlying myelosuppression and graft failure are poorly understood. Early studies using in vitro systems indicated that HCMV infection of CD34^+^ hematopoietic progenitor cells (HPCs) alters myeloid development [2,3,4,5]. We recently published that latent HCMV infection of CD34^+^ HPCs induces the secretion of TGF-β that is responsible for virus-mediated myelosuppression [6]. Even though these results provide relevant insights into the mechanism(s) for how the latent HCMV infection of a minor population of CD34^+^ HPCs in the bone marrow induces global myelosuppression in HSCT patients, the strict species specificity of HCMV and the lack of surrogate animal models have precluded the in vivo validation of these findings as well as the development of preventive strategies that can target latently infected cells.

Generation of humanized mice has been instrumental for the direct in vivo investigation of viruses with growth restricted to human cells. Our group has developed the first humanized mouse model in which human CD34^+^ HPC-engrafted NOD.Cg-Prkdc^scid^*IL2rγ*^tm1Wjl^ null (huNSG) mice infected with HCMV can support a latent viral infection and where reactivation occurs in human macrophages following G-CSF-induced mobilization of bone-marrow hematopoietic cells [7]. These data recapitulate observations made in hematopoietic stem cell transplant patients receiving G-CSF-mobilized cells from HCMV-seropositive donors [8,9,10] and also provide definitive evidence that CD34^+^ HPCs and monocytes are the source of latent HCMV (reviewed in [11]). Recently, we have extended this HCMV humanized mouse model by utilizing NSG mice implanted with fetal bone marrow, liver, and thymus (huBLT mice). Similar to the huNSG model, we have shown that HCMV establishes latency and reactivates upon administration of G-CSF [12]. The huBLT mouse model is superior to conventional humanized mouse models since engraftment of human thymic tissue in these mice supports development of a functional adaptive immune system including functional CD4^+^ and CD8^+^ T-cells as well as B-cells with improved class switching [13]. Indeed, we and others have previously demonstrated that the huBLT model supports HCMV-specific T-cell and antibody responses [12,14]. Finally, huBLT mice develop more mature cell types including fibroblasts, endothelial, and epithelial cells and develop a mature bone marrow environment allowing the investigation of human hematopoiesis. 

In the present study, we developed a novel, modified HCMV-huBLT mouse model by engrafting the animals with a minor population of HCMV-infected progenitor cells in the context of a larger number of uninfected cells to mimic the common occurrence of a small number of CD34^+^ HPCs harboring HCMV in seropositive transplant donors. Here, we demonstrate evidence of HCMV-mediated engraftment failure after hematopoietic cell transplantation, thereby recapitulating the clinical observations and validating this model as a tool for testing novel therapeutics.

## 2. Materials and Methods 

### 2.1. CD34^+^ HPC Isolation

CD34^+^ hematopoietic progenitor cells (HPCs) were isolated from de-identified human fetal liver obtained from Advanced Bioscience Resources using CD34 magnetic bead separation as previously described [12]. Briefly, a single cell suspension from total liver tissue (17 wks) was generated using mechanical and enzymatic disruption followed by RBC lysis and Ficoll gradient purification. CD34^+^ HPCs were enriched using the CD34 micro-bead isolation kit from Miltenyi Biotech, and purity was verified by flow cytometry analysis to be greater than 85%. 

### 2.2. CD34^+^ HPC Colony Formation Assay

Primary CD34^+^ HPCs were thawed (with an average viability of 80% post-thaw) and recovered overnight in stem cell media. HPCs were then infected with HCMV, FACS-isolated, and plated for analysis of colony formation ability as previously described [6]. CD34^+^ HPCs were infected with wild-type HCMV (strain: TB40/E-GFP) or mock-infected for 24–48 hrs. Following infection, a pure population of viable CD34^+^, GFP^+^ (HCMV) or viable CD34^+^ (Mock) HPCs were isolated by FACS using a BD FACS Aria equipped with 488, 633, and 405 nm lasers, running FACS Diva. Sorted HPCs were plated in Methocult H4434 (Stem Cell Technologies) in 35 mm dishes in triplicate for myeloid colony assays. Total and specific colonies were enumerated manually at 14 days post-plating using a standard microscope. Experiments were performed at least in triplicate. 

### 2.3. Mice

NOD.Cg-Prkdc^scid^*IL2rγ*^tm1Wjl^ (NSG) mice were purchased from Jackson Laboratories and bred in-house. All mice were housed in micro-isolator cages in a designated specific pathogen-free facility at OHSU and fed sterile food and water ad litem. Euthanasia was performed via administration of CO_2_ according to the American Veterinary Medical Association guidelines (2020 Edition). All animal experiments were conducted under the approved OHSU Institutional Animal Care and Use Committee protocol 0922, and mice were managed in accordance with the NIH Office of Laboratory Animal Welfare: “PHS Policy on the Humane Care and Use of Research Animals” (Revised 2015)) and the recommendations of the AAALAC: “The Guide for the Care and Use of Laboratory Animals, 8th edition” (2011 Edition)

### 2.4. Generation of HuBLT Mice

Humanized bone-marrow–liver–thymus mice were generated as previously described [12]. Briefly, adult NSG mice were anesthetized, and ~1 mm pieces of human fetal thymus and liver tissue were surgically implanted under the right kidney capsule. At one week post-surgery, mice were sub-lethally irradiated with 200 cGy using a ^137^Cs gamma radiation source. At 24 h post-irradiation, mice were transplanted with 4.2 × 10^5^ autologous CD34^+^ HPCs via intravenous injection. To generate HCMV-infected huBLT mice, a portion of autologous CD34^+^ HPCs were infected with wild-type HCMV (strain: TB40/E-GFP) as described above for 24 hrs, then FACS-isolated for a pure population of viable CD34^+^, GFP^+^ HPCs or control mock-infected viable CD34^+^ HPCs. These cells were then mixed at a ratio of 1:70 with bulk autologous CD34^+^ HPCs prior to injection. 

### 2.5. Analysis of Human Cell Engraftment

Beginning at 2 weeks post-HPC transplant, mice were screened for human cell engraftment by peripheral tail-vein bleed and flow cytometry analysis. Freshly isolated mononuclear cells from blood, bone marrow, or spleen from huBLT or control mice were analyzed by flow cytometry at the indicated times as previously described [12]. Briefly, non-specific binding was blocked prior to and during staining using human and mouse serum, and viability was determined by staining with ZombieAqua viability dye (Biolegend) prior to antibody staining. Samples were stained using antibodies specific for human cell surface markers: CD3 (clone UCHT1), CD4 (OKT4), CD8 (HIT8a), CD11c (3.9), CD14 (HCD14), CD19 (HIB19), CD20 (2H7), CD29 (TS2/16), CD31 (WM59), CD34 (581), CD45 (HI30); and murine cell surface marker CD45 (30-F11), all from Biolegend. All samples were fixed in 2% neutral buffered formalin prior to analysis using an LSRII flow cytometer equipped with FACS Diva (Becton Dickson). Post-acquisition analysis was performed using FlowJo v10 (TreeStar) with gates for human cell populations set using an equivalent stained sample from a non-humanized control NSG mouse, and gates for murine cell populations were set using an equivalent stained sample of human lymphocytes.

## 3. Results and Discussion

**Figure 1:**
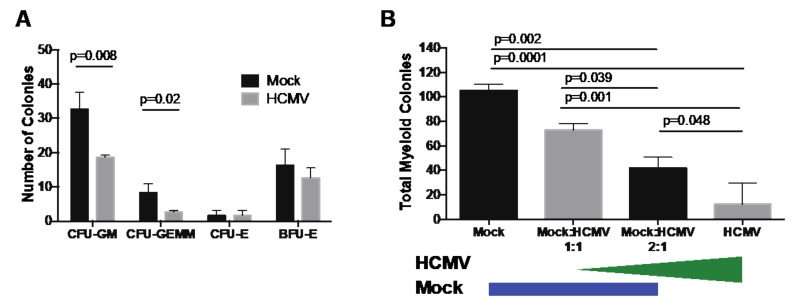
Human cytomegalovirus (HCMV) directly and indirectly suppresses myelopoiesis in vitro. CD34^+^ hematopoietic progenitor cells (HPCs) were infected with HCMV (TB40E/GFP) for 24–48 hrs. Infected cells were FACS-isolated for viable, CD34^+^, GFP^+^ HPCs. Uninfected cells were cultured in the same manner and sorted for viable CD34^+^ HPCs. (**A**) For analysis of the direct effect of HCMV infection on myeloid differentiation, sorted CD34^+^ (Mock) or CD34^+^GFP^+^ (HCMV) HPCs were plated at 500 cells per dish in Methocult H4434 and cultured for 14 days. Different myeloid (CFU-GM and CFU-GEMM) and erythroid (CFU-E and BFU-E) colonies were counted manually at 14 days post-plating. A representative experiment is shown in A. (**B**) For analysis of the indirect effect of HCMV infection on myeloid differentiation, sorted CD34^+^ (Mock) or CD34^+^GFP^+^ (HCMV) HPCs were plated at 500 cells per dish, or mixed at a ratio of 1:1 Mock:HCMV (500 Mock plus 500 HCMV), or at a ratio of 2:1 Mock:HCMV (500 Mock plus 250 HCMV), then plated in Methocult H4434. Total colonies were counted manually at 14 days post-plating. A representative experiment is shown in B. Error bars represent standard deviation for replicate dishes. Significance determined by unpaired t-test with Holm–Sidak correction for each colony type (**A**) or one-way ANOVA with Tukey correction (**B**), and *p*-values are listed for significant groups.

Previous studies have shown that direct HCMV infection of CD34^+^ HPCs inhibits myeloid differentiation and proliferation [2,3,4,5]. Additional data also support the role of the classical myeloid colony formation assay as a predictive measure of HSPC transplant success [15,16]. Therefore, to confirm HCMV inhibition of myelopoiesis in CD34^+^ HPCs used in this study, myeloid colony-forming assays were used to quantitate formation of granulocyte–macrophage (CFU-GM), granulocyte–erythroid–macrophage–megakaryocyte (CFU-GEMM), erythroid (CFU-E) and burst-forming erythroid (BFU-E) colonies. Briefly, CD34^+^ HPCs were infected with HCMV strain TB40/E (MOI 3) purified by FACS for CD34^+^GFP^+^ HPCs and cultured for 14 days in Methocult H4434. As shown in Figure 1A, HCMV infection suppressed myeloid (CFU-GM reduced by 43%, and CFUGEMM reduced by 78%) but not erythroid (CFU-E and BFU-E) colony formation compared to mock, which is comparable with previously published results from our laboratory and others [2,3,4,5,6]. To determine the indirect effects of HCMV-infected cells on the myelopoiesis potential of uninfected cells, such as occurs during transplant, HCMV-GFP infected CD34^+^ HPCs were added to uninfected cells at a ratio of 1:1 or 1:2 and plated on Methocult. As shown in Figure 1B, co-culture of HCMV-infected HPCs suppresses myelopoiesis of neighboring uninfected HPCs in a dose-dependent manner (31%, 60%, and 88% reduction compared to Mock, respectively).

**Figure 2:**
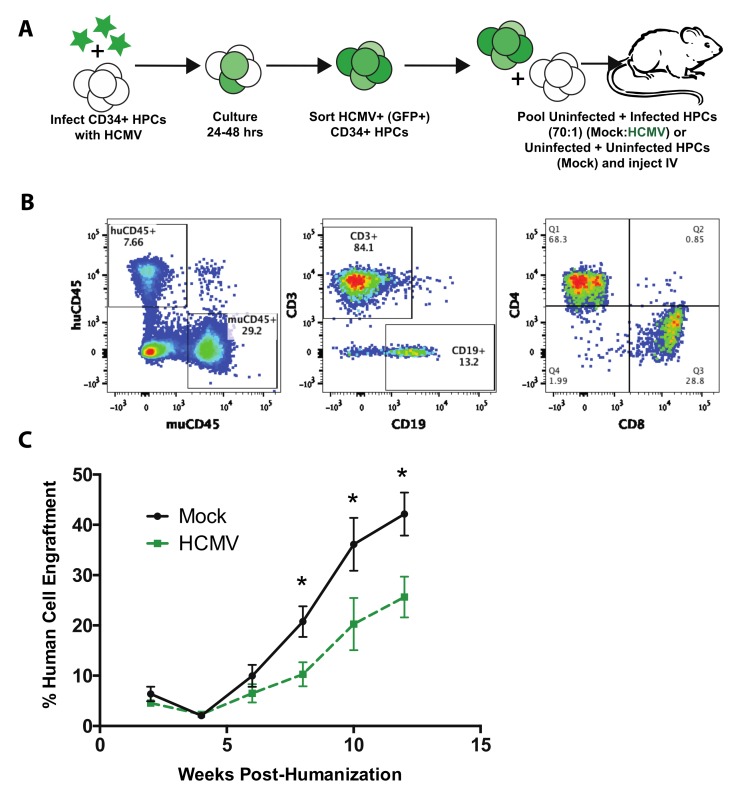
HCMV infection suppresses CD34^+^ HPC engraftment in huBLT mice. (**A**) huBLT (bone-marrow–liver–thymus)-NSG mice were generated by transplantation of human fetal liver and thymus under the kidney capsule of adult NSG mice. Post-surgical transplant, mice were sub-lethally irradiated and intravenously (IV) injected with autologous human CD34^+^ hematopoietic progenitor cells (HPCs). All huBLT mice were IV-injected with autologous human CD34^+^ HPCs at 4.2e5 cells per mouse. HCMV-infected huBLT mice were generated by pre-infection of a subset of CD34^+^ HPCs with wild-type HCMV (TB40/E-GFP) for 24 hrs. Infected cells were sorted by FACS for viable, CD34^+^GFP^+^ cells and mixed with uninfected cells (at a 1:70 ratio: 6e3 infected HPCs plus 4.2e5 uninfected HPCs per mouse) prior to injection (HCMV). A control population was mock-infected and sorted in the same manner to generate the uninfected group (Mock). *n* = 7 per group. **(B)** Mice were bled every two weeks post-humanization and screened for human cell engraftment by flow cytometry using antibodies specific for huCD45, huCD3, huCD19, huCD4, huCD8, and muCD45. A representative flow cytometry gating strategy is shown. Panel 1: huCD45 vs. muCD45 leukocytes (gated on total viable leukocytes); Panel 2: CD3^+^ T-cells vs. CD19^+^ B-cells (gated on huCD45^+^ cells in panel 1); Panel 3: huCD4^+^ and huCD8^+^ T-cells (gated on huCD3^+^ T-cells in panel 2). Data shown are of an uninfected (Mock) mouse at 10 weeks post-humanization. (**C**) Time-course of human CD45^+^ leukocyte engraftment overtime. Data shown are the average of the total percentage of huCD45^+^ lymphocytes and monocytes out of all CD45^+^ lymphocytes and monocytes (huCD45^+^ plus mouse CD45^+^), gated on viable cells for each group. Error bars represent standard error of the mean, *: *p* < 0.05.

Small animal models, such as mice, have been extensively used to study human disease and to develop new therapeutic interventions. Humanized mice provide a useful tool to examine mechanisms of disease caused by human pathogens that do not have animal models. To assess the role of HCMV on human stem cell transplant, we modified our previously described huBLT mouse model of HCMV infection. In this study, adult NOD.Cg-Prkdc^scid^*IL2rγ*^tm1Wjl^ (NSG) mice were transplanted with human fetal liver and thymic tissue under the kidney capsule. One week after transplant, the mice were sub-lethally irradiated and injected intravenously with a mixture of autologous mock-infected or HCMV-infected human CD34^+^ HPCs (Figure 2A) to mimic an HCMV-seropositive bone marrow transplant. A fraction of the CD34^+^ HPC pool was pre-infected with HCMV (TB40/E-GFP) for 24 h, sorted for viable, CD34^+^GFP^+^ cells and then mixed with uninfected cells at a ratio of 1 infected to 70 uninfected HPCs, resulting in a final transplant wherein only 1.5% of the CD34^+^ HPC population was HCMV-infected. This ratio was chosen based on previously reported clinical data. Ex vivo analysis suggests that between 0.004% and 0.01% of total bone marrow mononuclear cells harbor HCMV [17], although the CD34^+^-enriched fraction, using comparable techniques, suggests an HCMV infection rate of between 0.01% and 1% [18,19,20,21]. 

As we previously reported in huNSG mice, the virus establishes a latent infection over a period of 4–8 weeks [7], resulting in viral genome levels below the limit of detection (data not shown). This result is in accordance with the low rate of viral reactivation reported in HCMV-seropositive patients undergoing autologous bone marrow transplant [22,23].

To monitor engraftment and maturation of HPCs, mice were bled every 2 weeks post-humanization and screened for human cell engraftment by flow cytometry using antibodies specific for human CD45 and mouse CD45 (Figure 2B, panel 1). The engrafting human cell population in these mice was further refined by flow cytometry analysis of the major lymphoid subsets including B-cells (CD19), T-cells (CD3), and T-cell subsets (CD4 vs. CD8) (Figure 2B, Panels 2 and 3). Total human cell engraftment was measured as the percentage of human CD45^+^ leukocytes out of total leukocytes (human CD45^+^ plus mouse CD45^+^) in the periphery. As shown in Figure 2C, human cell engraftment in uninfected humanized mice increased over time. However, when an initial subset of the transplanted HPC population was HCMV-infected, engraftment was significantly suppressed (up to 50%) starting at 8 weeks post-transplantation. 

**Figure 3:**
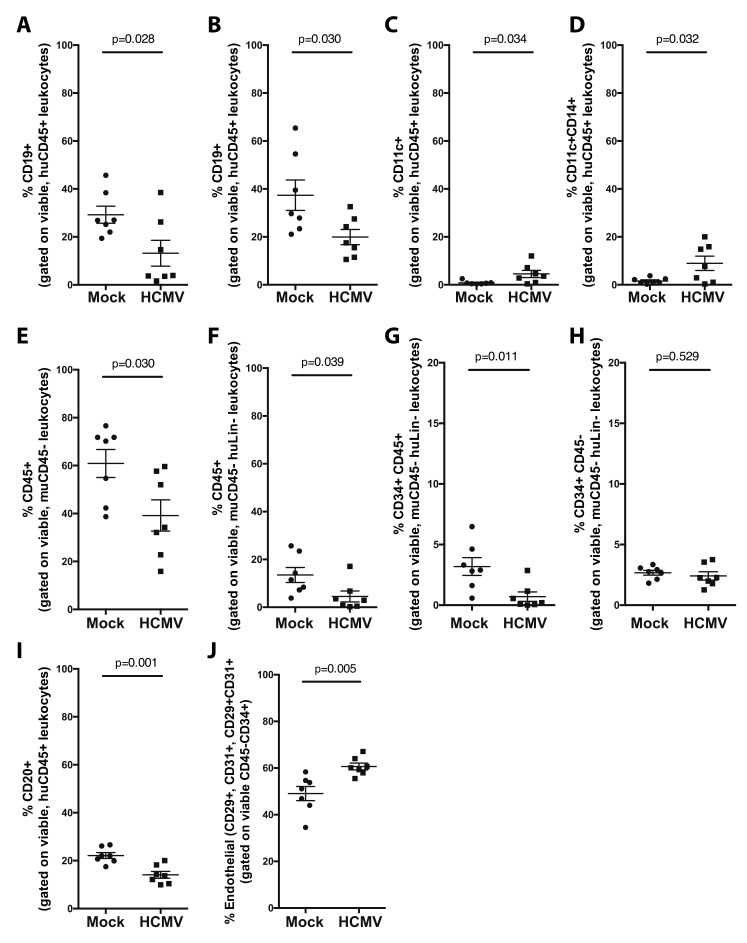
HCMV preferentially alters CD34^+^ HPC engraftment and differentiation of myeloid and lymphoid populations in vivo in huBLT mice. huBLT mice were generated and infected with HCMV as described in Figure 1. Analysis of the mature lymphoid and myeloid populations in the periphery at 12 weeks post-humanization was performed by flow cytometry analysis of total viable muCD45^−^ leukocytes. Analysis of the mature, peripheral populations was performed by quantification of CD19^+^ B-cells (gated on huCD45^+^ leukocytes) in the blood (**A**) and spleen (**B**); and CD11c^+^ dendritic cells (**C**) or CD11c^+^CD14^+^ bulk monocytic cells (**D**) (gated on huCD45^+^ viable leukocytes) in the spleen. Analysis of engraftment success and stem cell maturation was performed on total bone marrow flushed from the femur by quantification of the following: total human leukocytes (gated on huCD45^+^, muCD45^−^, viable leukocytes) (**E**), bulk hematopoietic progenitors (gated on huCD45^+^, muCD45^−^, huLin-, viable leukocytes) (**F**), CD34^+^ lymphoid and myeloid progenitor cells (gated on huCD45^+^, muCD45^−^, huLin^-^, viable leukocytes) (**G**); and CD34^+^ HPCs (gated on huCD45^−^, muCD45^−^, huLin^-^, viable leukocytes) (**H**). Quantification of specific cell populations in the bone marrow: total CD20^+^ B-cells (gated on huCD45^+^ viable leukocytes) (**I**) and total hemato-endothelial progenitors (gated on huCD29^+^ and/or huCD31^+^, huCD45^−^, huCD34^+^ viable leukocytes) (**J**). All data show standard error of the mean for 7 individual mice per group. Significance was determined by unpaired t-test, and *p*- values are listed.

Analysis of lymphoid tissues at 12 weeks post-humanization was performed by flow cytometry analysis to determine if a minor subset of HCMV-infected HPCs at the time of engraftment alters HPC differentiation within the engrafting human cell population. Within the human cell population in the periphery (blood and spleen, Figure 3A–D), HCMV differentially regulates the engraftment of human immune cell populations. Immune regulatory B-cell populations that are anti-viral, including CD19^+^ B-cells circulating in the periphery (Figure 3A) and resident in the spleen (Figure 3B), were decreased 2-fold in both compartments. In contrast, cellular populations beneficial for viral replication, including CD11c^+^ dendritic cells (Figure 3C) and CD14^+^CD11c^+^ monocytes (Figure 3D), were increased. These hematopoietic changes are within the background of overall suppression of total human cell engraftment in lymphoid compartments.

Since manipulation of progenitor cell populations is important for HCMV regulation of myelopoiesis in vitro, we also assessed the effect of this small population of HCMV-infected HPCs on the overall engraftment and differentiation of human cell populations in the bone marrow of humanized mice. We observed that total human CD45^+^ leukocytes (Figure 3E), lineage negative (Lin^-^) CD45^+^ leukocytes (Figure 3F), and Lin^-^CD34^+^CD45^+^ hematopoietic progenitor cells (Figure 3G) were suppressed in the bone marrow. However, the earlier hematopoietic progenitor cell population (CD34^+^CD45^−^) was unchanged (Figure 3H), suggesting a defect in differentiation or proliferation of leukocytes or hematopoietic-committed progenitors rather than a defect in initial seeding of the bone marrow in vivo. In addition, as seen in the periphery, immune populations with anti-viral effect, including CD20^+^ B-cells, were decreased (Figure 3I). Indeed, a study by Florescu et al. reported that early hypogammaglobinemia (≤1 month post-transplant) is observed in thoracic transplant recipients and is associated with a dramatic increase in the risk of CMV infection [24]. Finally, we observed an increase in endothelial progenitors, defined here as CD45^−^CD34^+^ progenitors, that also express either or both of the hemato-endothelial cell markers CD29 or CD31 (Figure 3J), confirming the important role played by endothelial cells in establishing a persistent long-term productive infection [25]. 

Overall, we conclude that latent HCMV infection of CD34^+^ HPCs alters the bone marrow environment such that the virus promotes the differentiation of myeloid cells but inhibits lymphoid blood cell production in the periphery of humanized mice. This work complements and expands on our previous study using huBLT mice as a model of HCMV latency and reactivation in contest of a functional immune system [12]. This new HCMV-huBLT mouse model provides an ideal system to examine the effects of different viral genes on hematopoiesis that are generally too complex for tissue culture-based systems and impossible to experimentally approach in human patients. We recently published that both HCMV UL7 and US28 are required for the expansion of the CD14^+^ monocyte population in the huNSG model [26,27]. This novel model system will provide a tractable in vivo system in which to determine the effect of these and other viral genes during HSCT. Thus, this advanced HCMV-huBLT mouse model provides a unique opportunity to gain further insight into the fundamental mechanisms of HCMV myelosuppression after HSCT and will be extremely useful to test novel therapeutic strategies.

## References

[1] Boeckh M., Geballe A.P. (2011). Cytomegalovirus: pathogen, paradigm, and puzzle. J. Clin. Investig..

[2] Rakusan T.A., Juneja H.S., Fleischmann W.R. (1989). Inhibition of hemopoietic colony formation by human cytomegalovirus in vitro. J. Infect. Dis..

[3] Maciejewski J.P., Bruening E.E., Donahue R.E., Mocarski E.S., Young N.S., St Jeor S.C. (1992). Infection of hematopoietic progenitor cells by human cytomegalovirus. Blood.

[4] Goodrum F., Jordan C.T., Terhune S.S., High K., Shenk T. (2004). Differential outcomes of human cytomegalovirus infection in primitive hematopoietic cell subpopulations. Blood.

[5] Sing G.K., Ruscetti F.W. (1990). Preferential suppression of myelopoiesis in normal human bone marrow cells after in vitro challenge with human cytomegalovirus. Blood.

[6] Hancock M.H., Crawford L.B., Pham A.H., Mitchell J., Struthers H.M., Yurochko A.D., Caposio P., Nelson J.A. (2020). Human Cytomegalovirus miRNAs Regulate TGF-beta to Mediate Myelosuppression while Maintaining Viral Latency in CD34(+) Hematopoietic Progenitor Cells. Cell Host Microbe.

[7] Smith M.S., Goldman D.C., Bailey A.S., Pfaffle D.L., Kreklywich C.N., Spencer D.B., Othieno F.A., Streblow D.N., Garcia J.V., Fleming W.H. (2010). Granulocyte-colony stimulating factor reactivates human cytomegalovirus in a latently infected humanized mouse model. Cell Host Microbe.

[8] Anderson D., DeFor T., Burns L., McGlave P., Miller J., Wagner J., Weisdorf D. (2003). A comparison of related donor peripheral blood and bone marrow transplants: Importance of late-onset chronic graft-versus-host disease and infections. Biol. Blood Marrow Transplant..

[9] Blaise D., Kuentz M., Fortanier C., Bourhis J.H., Milpied N., Sutton L., Jouet J.-P., Attal M., Bordigoni P., Cahn J.-Y. (2000). Randomized Trial of Bone Marrow Versus Lenograstim-Primed Blood Cell Allogeneic Transplantation in Patients With Early-Stage Leukemia: A Report From the Société Française de Greffe de Moelle. J. Clin. Oncol..

[10] Champlin R.E., Schmitz N., Horowitz M.M., Chapuis B., Chopra R., Cornelissen J.J., Gale R.P., Goldman J.M., Loberiza F.R.J., Hertenstein B. (2000). Blood stem cells compared with bone marrow as a source of hematopoietic cells for allogeneic transplantation. IBMTR Histocompatibility and Stem Cell Sources Working Committee and the European Group for Blood and Marrow Transplantation (EBMT). Blood.

[11] Goodrum F. (2016). Human Cytomegalovirus Latency: Approaching the Gordian Knot. Annu. Rev. Virol..

[12] Crawford L.B., Tempel R., Streblow D.N., Kreklywich C., Smith P., Picker L.J., Nelson J.A., Caposio P. (2017). Human Cytomegalovirus Induces Cellular and Humoral Virus-specific Immune Responses in Humanized BLT Mice. Sci. Rep..

[13] Covassin L., Jangalwe S., Jouvet N., Laning J., Burzenski L., Shultz L.D., Brehm M.A. (2013). Human immune system development and survival of non-obese diabetic (NOD)-scid IL2rgamma(null) (NSG) mice engrafted with human thymus and autologous haematopoietic stem cells. Clin. Exp. Immunol..

[14] Wahl A., De C., Abad Fernandez M., Lenarcic E.M., Xu Y., Cockrell A.S., Cleary R.A., Johnson C.E., Schramm N.J., Rank L.M. (2019). Precision mouse models with expanded tropism for human pathogens. Nature Biotechnol..

[15] Page K.M., Zhang L., Mendizabal A., Wease S., Carter S., Gentry T., Balber A.E., Kurtzberg J. (2011). Total Colony-Forming Units Are a Strong, Independent Predictor of Neutrophil and Platelet Engraftment after Unrelated Umbilical Cord Blood Transplantation: A Single-Center Analysis of 435 Cord Blood Transplants. Biol. Blood Marrow Transplant..

[16] Prasad V.K., Mendizabal A., Parikh S.H., Szabolcs P., Driscoll T.A., Page K., Lakshminarayanan S., Allison J., Wood S., Semmel D. (2008). Unrelated donor umbilical cord blood transplantation for inherited metabolic disorders in 159 pediatric patients from a single center: influence of cellular composition of the graft on transplantation outcomes. Blood.

[17] Slobedman B., Mocarski E.S. (1999). Quantitative analysis of latent human cytomegalovirus. J. Virol..

[18] Sindre H., Tjoonnfjord G.E., Rollag H., Ranneberg-Nilsen T., Veiby O.P., Beck S., Degre M., Hestdal K. (1996). Human cytomegalovirus suppression of and latency in early hematopoietic progenitor cells. Blood.

[19] Goodrum F.D., Jordan C.T., High K., Shenk T. (2002). Human cytomegalovirus gene expression during infection of primary hematopoietic progenitor cells: a model for latency. Proc. Natl. Acad. Sci. USA.

[20] Mendelson M., Monard S., Sissons P., Sinclair J. (1996). Detection of endogenous human cytomegalovirus in CD34+ bone marrow progenitors. J. Gen. Virol..

[21] Von Laer D., Meyer-Koenig U., Serr A., Finke J., Kanz L., Fauser A.A., Neumann-Haefelin D., Brugger W., Hufert F.T. (1995). Detection of cytomegalovirus DNA in CD34+ cells from blood and bone marrow. Blood.

[22] Verdonck L., de Gast G., van Heugten H., Nieuwenhuis H., Dekker A. (1991). Cytomegalovirus infection causes delayed platelet recovery after bone marrow transplantation. Blood.

[23] Torok-Storb B., Simmons P., Khaira D., Stachel D., Myerson D. (1992). Cytomegalovirus and marrow function. Ann. Hematol..

[24] Florescu D.F., Kalil A.C., Qiu F., Schmidt C.M., Sandkovsky U. (2013). What Is the Impact of Hypogammaglobulinemia on the Rate of Infections and Survival in Solid Organ Transplantation? A Meta-Analysis. Am. J. Transplant..

[25] Jarvis M.A., Nelson J.A. (2007). Human cytomegalovirus tropism for endothelial cells: not all endothelial cells are created equal. J. Virol..

[26] Crawford L.B., Kim J.H., Collins-McMillen D., Lee B.J., Landais I., Held C., Nelson J.A., Yurochko A.D., Caposio P. (2018). Human Cytomegalovirus Encodes a Novel FLT3 Receptor Ligand Necessary for Hematopoietic Cell Differentiation and Viral Reactivation. MBio.

[27] Crawford L.B., Caposio P., Kreklywich C., Pham A.H., Hancock M.H., Jones T.A., Smith P.P., Yurochko A.D., Nelson J.A., Streblow D.N. (2019). Human Cytomegalovirus US28 Ligand Binding Activity Is Required for Latency in CD34(+) Hematopoietic Progenitor Cells and Humanized NSG Mice. MBio.

